# Do Volume para a Geração de Valor em Cirurgia Cardíaca: O que Falta para Dar a Largada no Brasil?

**DOI:** 10.36660/abc.20230036

**Published:** 2023-02-13

**Authors:** Omar Asdrúbal Vilca Mejia, Fabio Biscegli Jatene

**Affiliations:** 1 Hospital das Clínicas Faculdade de Medicina Universidade de São Paulo São Paulo SP Brasil Instituto do Coração do Hospital das Clínicas da Faculdade de Medicina da Universidade de São Paulo, São Paulo, SP – Brasil

**Keywords:** Cirurgia Cardíaca, Banco de Dados, Gestão de Qualidade, Avaliação de Resultados em Cuidados de Saúde, Monitoramento de Resultados

*“A estrada que leva ao sucesso está sempre em construção”*

**Lily Tomlin**

Algumas características propiciaram o grande desenvolvimento da cirurgia cardiovascular, dentre elas a criatividade para o desenvolvimento de técnicas e dispositivos e principalmente muita coragem. Assim, ela teve uma evolução fantástica, movida pelo impacto que causaria na qualidade de vida dos pacientes, tornando-se rapidamente um dos procedimentos mais realizados no mundo.^1^ Talvez isto não teria acontecido se não fossem por dois fatores: por um lado a destreza técnica, produto do aumento do volume cirúrgico; e, por outro, as informações resultantes das análises de bancos de dados.^2 , 3^ Isto causou uma melhoria contínua das técnicas perioperatórias, dos dispositivos, e dos resultados.

Isto pode explicar a melhora dos desfechos, mesmo quando os procedimentos se tornaram mais complexos e em pacientes cada vez mais graves.^4^ A partir da análise destes registros surgiram algumas necessidades a serem resolvidas.^5^ Esta foi a época em que escores de predição de risco de mortalidade foram introduzidos, com a finalidade de ajustar e entender melhor os resultados. A partir disto, e para conseguir a melhoria contínua, a implementação de programas de qualidade se tornou uma necessidade.^6^ Outro passo importante foi também a iniciativa de tornar públicos os resultados dos hospitais, introduzindo o conceito da transparência.^7^

Entretanto outras variáveis entraram em cena, pois com o aumento da expectativa de vida, a indicação de cirurgia se tornou mais frequente em pacientes idosos e frágeis onde há um aumento da taxa de complicações, com um aumento dos tempos de internação e consequentemente dos custos.^8^ Isto passou a se tornar um problema para as fontes pagadoras, públicas ou privadas, onde otimizar se tornaria a única alternativa, principalmente em um ecossistema onde hospitais e funcionários, na sua grande maioria, são ressarcidos por meio de modelos de pagamento por serviço ( *fee for service* ). Isto remete a uma reflexão de um modelo que privilegia o conceito de que quanto mais intervenções ou mais tempo no hospital melhor, em um sistema que não necessariamente premia o melhor resultado. Assim hospitais com menos complicações poderiam ter menor ressarcimento e vice-versa.^9^ Portanto a sobrevivência deste modelo, mesmo com práticas adequadas, passou a ser questionada.^10^

Existem na atualidade mais de 50 modelos de pagamento, baseados em valor, que podem ser escolhidos pela fonte pagadora, já que variam em função dos riscos e dos tipos de reembolso.^11^ Na maior parte das vezes estes modelos são caracterizados por uma das alternativas a seguir: 1- redução dos gastos, sem perder a qualidade do atendimento; 2- melhorar a qualidade sem aumentar os gastos; ou, idealmente, 3- melhorar a qualidade e reduzir os gastos. Em alguns destes modelos, as cirurgias cardiovasculares são apenas um item dentro de um pacote de ressarcimento já atribuído ao respectivo grupo de diagnósticos relacionados (GDR). Portanto, os esforços para diminuir complicações e tempos de internação, após uma cirurgia cardiovascular, ajudariam na redução dos gastos do hospital. Dentre os vários modelos, há aqueles que se adaptam melhor à determinadas situações, mas independentemente disto, sabemos que gerar valor em saúde, sempre foca nos melhores resultados para os pacientes, e é aí onde os cirurgiões cardiovasculares podem ter um impacto significativo.

Mais recentemente exemplos de bons resultados cirúrgicos, com custo baixo, começaram a serem divulgados.^12^ Por meio de equipes bem treinadas se conseguiu implementar protocolos, otimizar processos, levando a menos complicações e, consequentemente, menor custo. Dentro disto, o que mais chamou a atenção foram os hospitais que ofereciam cirurgias cardíacas a custo baixo e com ótimos resultados.^13^ Pouco mais longe foi o plano de saúde Geisinger quando anunciou “cirurgia cardíaca com garantia ou seu dinheiro de volta”.^14^ Exemplos mais recentes, como o modelo do cuidado perfeito “ *Perfect Care* ” mostrou redução de 37% do custo, mantendo a qualidade do atendimento, quanto mais aderência às métricas de geração de valor.^15^ Na América Latina, exemplos iniciais de geração de valor em cirurgia cardiovascular mostraram uma redução da mortalidade, após o estabelecimento de métricas e a formação de núcleos de excêlencia^16^ e, por outro lado, uma diminuição significativa dos tempos de internação de forma segura e efetiva.^17^

Dentro disto, a diretriz do ERAS ( *Enhanced Recovery After Surgery* ) para cirurgia cardíaca publicada em 2019,^18^ consolidou um novo conceito que se fortificou após a chegada da COVID-19.^19^ Trata-se de protocolos perioperatórios, multiprofissionais e baseados em evidência, que buscaram melhorar resultados e diminuir os custos por meio da formação de uma linha de cuidado. Para isto acontecer em grande escala existiu a necessidade da implementação de bancos de dados, de educação e treinamento de equipes, com foco nestes princípios de melhoria contínua. Além disto é importante que estas ações tenham suporte de outras medidas como são as portarias nacionais do Medicare e Medicaid nos Estados Unidos que até 2030 se propõem a colocar todos seus pacientes em linhas de cuidados baseadas em valor.^20^ Mais recentemente o Ministério da Saúde do Brasil estabeleceu mudanças para um ressarcimento por resultados, através da portaria do QualiSUS-Cardio, introduzindo categorias baseadas em volume, mortalidade, tempo de internação e taxa de reinternação,^21^ o que cria um cenário promissor dentro destes princípios. Com certeza outras ações deverão se seguir, para reforçar este modelo de ressarcimento. Entretanto este modelo pode vir a criar distorções, que precisam ser bem equacionadas, pois pacientes de alto risco podem passar a ser recusados por alguns centros e encaminhados para outros serviços.^22^

Assim, dentro deste entendimento os resultados precisam ser ajustados e preparados para se tornarem públicos e transparentes. O ajuste de risco, portanto, é fundamental, pois conseguimos identificar que o custo para os cuidados de pacientes de baixo, médio e alto risco, era significativamente desproporcional por causa de diferenças entre as taxas de morbidade e mortalidade nos diferentes grupos de risco.^23^ A evolução desta portaria, em proposições futuras, poderia considerar categorias de desempenho para cada estrato de risco e não de forma geral. Como princípio, o escore de risco ideal deveria incluir características próprias do local. No estado de São Paulo temos o SPScore, um índice de risco criado com inteligência artificial e que poderia ajudar para um ressarcimento mais apropriado perante as fontes pagadoras, no nosso cenário.^24^

Podemos dizer que no Brasil, para acompanhar estas mudanças, existe no Departamento de Cardiopneumologia da Faculdade de Medicina da Universidade de São Paulo uma linha de pesquisa que começou em 2012 com o objetivo de estratificar e melhorar resultados em pacientes encaminhados para cirurgia cardiovascular. Alunos de graduação e pós-graduação pesquisam e publicam temas sobre implementação de grandes bancos de dados, construção de escores de risco utilizando inteligência artificial, identificação de estratégias custo-efetivas em grupos de risco e otimização de processos, entre outras. Ainda em elaboração estão projetos para treinamento de habilidades não técnicas e *coaching* cirúrgico para as equipes, assim como a disseminação de programas de melhoria em centros no país e na América Latina. Estratégias nacionais iniciais como a do pagamento por resultados fomentam a busca pela qualidade do atendimento, mas isto somente ganhará escala com o suporte de um grande banco de dados e especialistas capacitados em métricas para a geração de valor. Da nossa parte, buscamos colaborar com o novo ecossistema da saúde, desenvolvendo processos, treinando e capacitando equipes a aplicar os princípios da melhoria contínua, no fluxo de cuidados dos pacientes, para cirurgia cardiovascular.^25^
