# Supplementary material for: Do Volume para a Geração de Valor em Cirurgia Cardíaca: O que Falta para Dar a Largada no Brasil?
Source: Arq Bras Cardiol. 2023 Feb 13;120(2):e20230036. [Article in Portuguese] doi: 10.36660/abc.20230036 (PMC10263462; doi:10.36660/abc.20230036)
Supplement: Translation

# Translation: From Volume to Value Creation in Cardiac Surgery: What is Needed to Get off the Ground in Brazil?

**Authors:** Omar Asdrúbal Vilca Mejia, Fabio Biscegli Jatene

**Affiliations:** 1 Brasil Instituto do Coração do Hospital das Clínicas da Faculdade de Medicina da Universidade de São Paulo, São Paulo, SP – Brasil

**Keywords:** Cardiac Surgery, Database, Total Quality Management, Patient Centered Care, Outcome Assessment Health Care

*“The road to success is always under construction.”*

**Lily Tomlin**

Some characteristics favored the great development of cardiovascular surgery; among them, creativity for developing techniques and devices and, mainly, much courage. Thus, cardiovascular surgery had a fantastic evolution, driven by its impact on the patient’s quality of life, quickly becoming one of the most performed procedures in the world.^1^Maybe this would not have happened if not for two factors: on the one hand, the improvement of technical skills as a result of the increased surgical volume, and, on the other hand, the information from the databases analyzes.^2 , 3^This has led to continuous improvement in perioperative techniques, devices, and outcomes.

This may explain the outcome improvement, even when procedures have become more complex and performed in increasingly severe patients.^4^From the analysis of these records, some issues emerged to be resolved.^5^This was when mortality risk scores were introduced to adjust and better understand the results. From that moment, and to achieve continuous improvement, implementing quality programs has become a necessity.^6^Another important step was the hospitals’ public reporting initiatives, introducing the concept of transparency.^7^

However, other variables are involved since, with the increase in life expectancy, the indication for surgery has become more frequent in elderly and frail patients, thus increasing complication rate, hospital length of stay, and, consequently, costs.^8^This has become a problem for payment sources, public or private, and optimizing would be the only option, especially in a system where hospitals and employees, for the most part, are reimbursed through fee-for-service models. This leads to a reflection of a model that privileges the concept that the more interventions or, the longer the time in the hospital, the better, in a system that does not necessarily reward the best result. Thus, hospitals with fewer complication rates could have lower compensation and vice versa.^9^Therefore, the survival of this model, even with fair practices, started to be questioned.^10^

There are currently more than 50 value-based payment models, which can be chosen by the payment source, as they vary according to the risks and types of reimbursement.^11^Most of the time, these models are characterized by one of the following alternatives: 1- cost reduction, without losing the quality of care; 2- quality improvement without cost increase; or, ideally, 3- quality improvement and cost reduction. In some of these models, cardiovascular surgeries are just one item within a larger bundle already assigned to the respective diagnoses-related group (DRG). Therefore, efforts to reduce complications and length of stay after cardiovascular surgery would help reduce hospital costs. Among the models, some adapt better to certain situations, but regardless of this, we know that value creation in health always focuses on the best results for patients, and that is where cardiovascular surgeons can have a significant impact.

More recently, some examples of good surgical results at a low cost have begun to be announced.^12^With well-trained teams, it was possible to implement protocols and optimize processes, leading to fewer complications and, consequently, lower costs. What most drew attention were hospitals that performed heart surgeries at low cost and with excellent results.^13^Geisinger Health System, a hospital group in the United States, went further when it announced “heart surgery guaranteed or your money back,” a 90-day warranty for elective coronary artery bypass surgery (CABG).^14^More recent examples, such as the Perfect Care approach, showed a 37% cost reduction, maintaining the quality of care when adherence to value creation metrics was higher.^15^In Latin America, initial examples of value creation in cardiovascular surgery showed a reduction in mortality after establishing metrics and forming centers of excellence^16^and, simultaneously, a significant decrease in the length of hospital stay in a safe and effective way.^17^

The Enhanced Recovery After Surgery (ERAS) guideline for cardiac surgery, published in 2019,^18^consolidated a new concept strengthened after the COVID-19 outbreak.^19^These perioperative, multidisciplinary, and evidence-based protocols sought to improve results and reduce costs by creating a line of care. For this to happen on a large scale, databases, education, and team training focusing on continuous improvement principles must be implemented. In addition, these actions must be supported by other measures, such as the Medicare and Medicaid ordinances in the United States, which by 2030 propose to have all their patients in value-based lines of care.^20^More recently, the Brazilian Ministry of Health established changes to reimbursement based on results through the QualiSUS-Cardio ordinance, introducing categories based on surgical volume, mortality, length of stay, and readmission rate,^21^which creates a promising scenario within these principles. Other actions will certainly come to reinforce this reimbursement model. However, this model may create distortion, which needs to be corrected, as high-risk patients may be referred to other institutions.^22^

Thus, results need to be adjusted and prepared to be revealed in a public and transparent way. Risk adjustment is essential, as we identified that the cost of care for low-, medium- and high-risk patients were significantly disproportionate due to differences between morbidity and mortality rates in different risk groups.^23^In future propositions, this ordinance could consider performance categories for each risk stratum and not in general. As a principle, the ideal risk score should include local characteristics. In São Paulo state, we have the SPScore, an artificial intelligence-generated risk index that could help with a more appropriate reimbursement to payment sources in our scenario.^24^

In Brazil, to keep up with these changes, a line of research at the Department of Cardiopneumology of the University of São Paulo Medical School began in 2012 to stratify and improve results in patients referred for cardiovascular surgery. Undergraduate and graduate students research and publish topics on implementing large databases, constructing artificial intelligence-generated risk scores, identifying cost-effective strategies in risk groups, and process optimization, among others. Projects for training non-technical skills and surgical coaching for the teams, as well as the dissemination of improvement programs in centers in the country and Latin America, are still under development. Initial national strategies, such as payment by results, encourage the search for quality of care, but this will only be scaled with the support of a large database and specialists trained in value creation metrics. We seek to collaborate with the new health system by developing processes, training, and qualifying teams to apply the principles of continuous improvement, in the patient care flow, for cardiovascular surgery.^25^
